# Mitochondrial aminoacyl-tRNA synthetase disorders: an emerging group of developmental disorders of myelination

**DOI:** 10.1186/s11689-019-9292-y

**Published:** 2019-12-16

**Authors:** Amena Smith Fine, Christina L. Nemeth, Miriam L. Kaufman, Ali Fatemi

**Affiliations:** 1 Moser Center for Leukodystrophies at the Kennedy Krieger Institute, Baltimore, MD 21205 USA; 2 Department of Neurology and Developmental Medicine, Kennedy Krieger Institute, Baltimore, MD 21205 USA; 3 Department of Neurology, Johns Hopkins University School of Medicine, Baltimore, MD 21287 USA

**Keywords:** Leukodystrophy, Mitochondrial aminoacyl-tRNA synthetases, DARS2, LBSL, EARS2, LTBL AARS2, Ovario-leukodystropy

## Abstract

**Background:**

The mitochondrial aminoacyl-tRNA synthetase proteins (mt-aaRSs) are a group of nuclear-encoded enzymes that facilitate conjugation of each of the 20 amino acids to its cognate tRNA molecule. Mitochondrial diseases are a large, clinically heterogeneous group of disorders with diverse etiologies, ages of onset, and involved organ systems. Diseases related to mt-aaRS mutations are associated with specific syndromes that affect the central nervous system and produce highly characteristic MRI patterns, prototypically the *DARS2*, *EARS*, and *AARS2* leukodystrophies, which are caused by mutations in mitochondrial aspartyl-tRNA synthetase, mitochondria glutamate tRNA synthetase, and mitochondrial alanyl-tRNA synthetase, respectively.

**Body:**

The disease patterns emerging for these leukodystrophies are distinct in terms of the age of onset, nature of disease progression, and predominance of involved white matter tracts. In *DARS2* and *EARS2* disorders, earlier disease onset is typically correlated with more significant brain abnormalities, rapid neurological decline, and greater disability. In *AARS2* leukodystrophy cases reported thus far, there is nearly invariable progression to severe disability and atrophy of involved brain regions, often within a decade. Although most mutations are compound heterozygous inherited in an autosomal recessive fashion, homozygous variants are found in each disorder and demonstrate high phenotypic variability. Affected siblings manifest disease on a wide spectrum.

**Conclusion:**

The syndromic nature and selective vulnerability of white matter tracts in these disorders suggests there may be a shared mechanism of mitochondrial dysfunction to target for study. There is evidence that the clinical variability and white matter tract specificity of each mt-aaRS leukodystrophy depend on both canonical and non-canonical effects of the mutations on the process of mitochondrial translation. Furthermore, different sensitivities to the mt-aaRS mutations have been observed based on cell type. Most mutations result in at least partial retention of mt-aaRS enzyme function with varied effects on the mitochondrial respiratory chain complexes. In *EARS2* and *AARS2* cells, this appears to result in cumulative impairment of respiration. Mt-aaRS mutations may also affect alternative biochemical pathways such as the integrated stress response, a homeostatic program in eukaryotic cells that typically confers cytoprotection, but can lead to cell death when abnormally activated in response to pathologic states. Systematic review of this group of disorders and further exploration of disease mechanisms in disease models and neural cells are warranted.

## Background

**Fig. 1:**
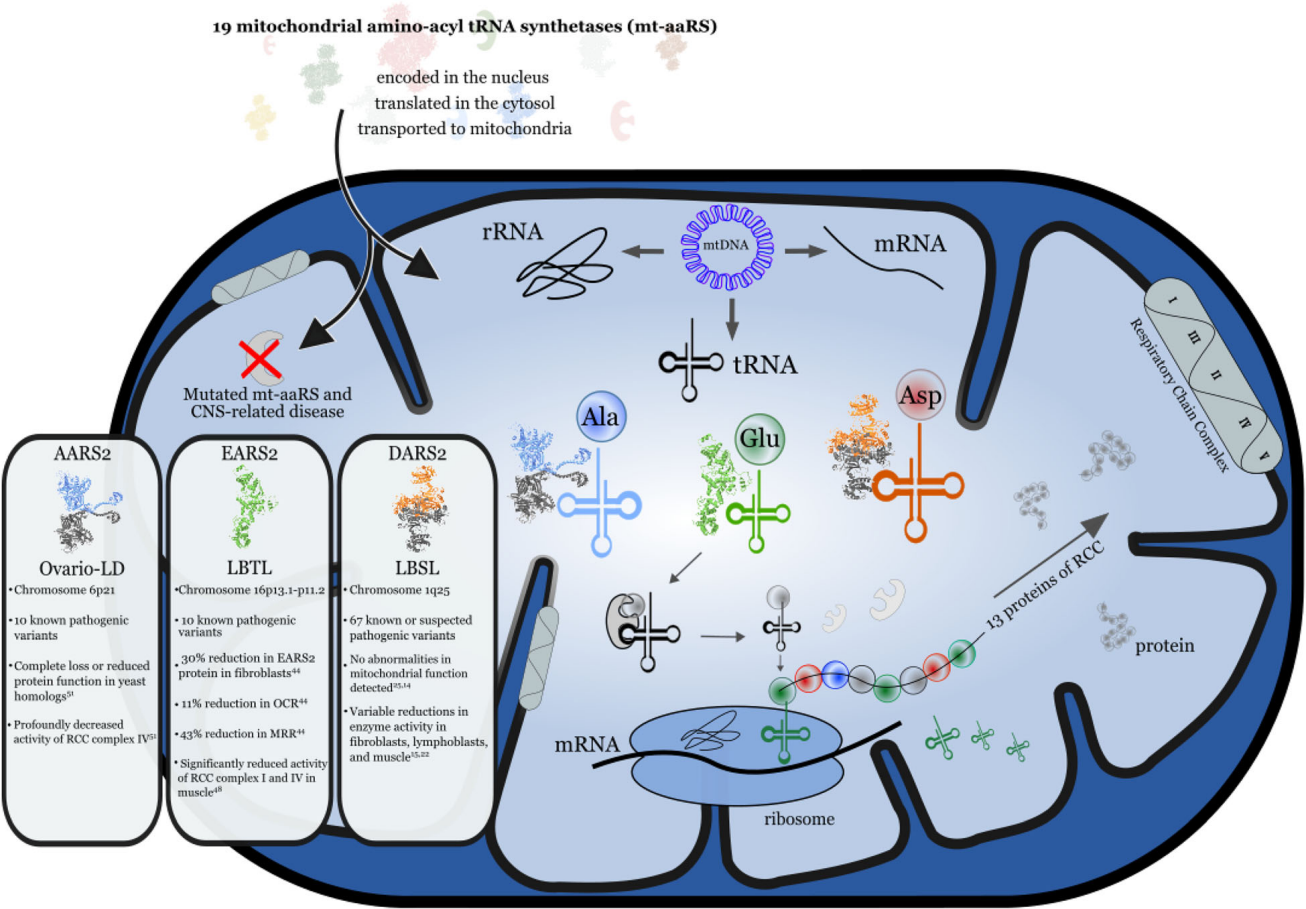
Effects of *AARS2*, *EARS2*, and *DARS2* mutations on mitochondrial translation and respiratory chain complex function. Human mt-aaRSs are encoded in the nucleus, synthesized in the cytosol, and delivered and imported into the mitochondria. To facilitate mitochondrial translation, the 19 mt-aaRSs catalyze the specific attachment of each amino acid onto the cognate tRNA(s). Specifically, AARS2 attaches alanine, EARS2 attaches glutamate, and DARS2 attaches aspartate. The molecular structures for AARS2, EARS2, and DARS2 are represented and the chromosomal location of the genes is listed. The EARS2 structure has not yet been determined. Mitochondrial translation synthesizes 13 proteins that, together with 84 additional nucleus-encoded proteins, form the five respiratory chain complexes. Thus, mt-aaRSs play a key role in cellular energy production, and mutations in mt-aaRSs often involve the central nervous system. In AARS-2 ovario-leukodystrophy (LD), LTBL, and LBSL, the effects of pathogenic variants at the cellular level are incompletely described. However, for all three disorders, there is a variable reduction but not complete absence of protein and reduced enzyme activity [6–10]. For AARS2 ovario-LD and LTBL, there is a subsequent RCC dysfunction, which has not yet been detected in LBSL patient cells [6–10]; oxygen consumption rate (OCR) and mitochondrial respiratory rate (MRR)

Mitochondria are comprised of products encoded by two genomes, nuclear and mitochondrial, totaling roughly 1500 genes [1, 2]. Aminoacyl-tRNA synthetase proteins (aaRSs) are a group of nuclear-encoded enzymes that ensure correct translation of the genetic code by conjugating each of the 20 amino acids to their cognate tRNA molecule [3–5]. The cytosolic aaRS enzymes supply aminoacyl-tRNA conjugates for protein translation, and the corresponding mt-aaRSs are imported into the mitochondrial matrix to perform their canonical role of charging amino acids to their mitochondrial genome-encoded tRNA molecules (mt-tRNA) (Fig. 1). Diseases of mitochondria, the powerplants of the cell, are a large and clinically heterogeneous group of disorders, encompassing a wide diversity of etiologies, ages of onset, involved organ systems, and clinical presentations. Disorders caused by mt-aaRS mutations are generating particular interest among mitochondrial diseases due to their predilection for the central nervous system (CNS) damage [11, 12]. It is well known that mitochondrial dysfunction preferentially affects high-energy demand tissues, especially the brain, muscle, and heart. Remarkably, nearly all mt-aaRS mutations result in CNS pathologies, including encephalopathies, Perrault syndrome, and leukodystrophies [5] (Table 1). Three mutations result in rare, well-defined leukodystrophy syndromes, namely, DARS2, caused by mutations in mitochondrial aspartyl-tRNA synthetase; EARS2, caused by mutations in mitochondria glutamate tRNA synthetase; and AARS2, caused by mutations in mitochondrial alanyl-tRNA synthetase. Only three mt-aaRS mutations lead to pathology outside the CNS. A comprehensive, dynamic source has been developed for emerging information about the mt-aaRS enzymes called MiSynPat (mitochondrial aminoacyl-tRNA synthetases and pathologies). This is a continuously updating database and web server that collects existing and emerging data related to mutations affecting the human mt-aaRSs [5].

**Table 1:** Neurological disorders associated with mt-aaRS mutations

Leukodystrophy	
DARS2	Leukoencephalopathy with brainstem and spinal cord involvement (LBSL)
EARS2	Leukoencephalopathy with thalamus and brainstem involvement and high lactate (LTBL)
AARS2	Ovario-leukodystrophy; rapid development of motor, cognitive, and psychiatric dysfunction
MARS2	Skeletal dysplasia, infantile cataracts, congenital neurotrophic keratitis, orbital myopathy, Leigh syndrome
Encephalopathy	
RARS	Pontocerebellar hypoplasia type 6
VARS2	Mitochondrial encephalomyopathy; psychomotor delay, epilepsy, intellectual disability, growth hormone deficiency, hyogonadism
WARS2	Autosomal recessive intellectual disability Mitochondrial encephalopathy Infantile-onset Parkinsonism
TARS2	Mitochondrial encephalomyopathy Axial hypotonia and limb hypertonia, psychomotor delay, and high levels of blood lactate
FARS2	Alpers sydnome, encephalopathy, epilepsy, lactic acidosis, spastic paraplegia
Perrault syndrome	
LARS2	Perrault syndrome—sensorineural deafness, ovarian abnormality, cognitive impairment, areflexia, dysarthia, and hyporeflexia
HARS2	Perrault syndrome
PARS2	Non-syndromic deafness, Leigh syndrome, intellectual disability with epilepsy and severe myopathy
NARS2	Non-syndromic deafness, Leigh syndrome, Alpers syndrome, infantile onset neurodegenerative disorder

Table 1 lists a brief, categorized summary of the neurologic disease phenotypes caused by mt-aaRS mutations.

As diseases associated with mt-aaRS enzymes are being further characterized, each appears to be associated with a very specific clinical syndrome. This phenomenon is best illustrated by the leukodystrophies caused by *DARS2* (leukoencephalopathy with brainstem and spinal cord involvement and high lactate, LBSL), *EARS2* (leukoencephalopathy with thalamus and brainstem involvement and high lactate, LTBL), and *AARS2* mutations (ovario-leukoencephalopathy). Mutations in *MARS2* are emerging as a fourth leukodystrophy, although only three cases have been reported thus far, in association with leukoencephalopathy, ataxia, and neurodevelopmental delays [13, 14].

The mt-aaRS disorders are inherited in an autosomal recessive fashion, and no de novo cases have been reported in the literature. The mechanisms underlying the specific genetic-clinical correlates are presently unknown. However, the clear syndromic nature of these disorders suggests there may be a shared mechanism that could be targeted for study. There is evidence that neuron-specific differences in the sensitivity to mutations may at least partially explain the selective vulnerability of specific white matter tracts in mt-aaRS leukodystrophies [15–17]. Furthermore, the pathophysiology of the mt-aaRS disorders reflects inconsistent biochemical alterations in the mitochondrial pathway, resulting in at least partial retention of enzyme function (Fig. 1) [6, 18]. We propose that systematic review of these disorders will identify patterns and information of prognostic value that will be applicable to mt-aaRS-related disorders that are more recently emerging.

## LBSL

Leukoencephalopathy with brainstem and spinal cord involvement and lactate elevation (LBSL) is typically characterized by slowly progressive gait difficulty secondary to spasticity, ataxia, and proprioceptive deficits with clear neuroimaging correlates in the pyramidal tracts, cerebellum, and dorsal columns [19, 20]. The hallmark diagnostic features are identification of a *DARS2* mutation, which encodes mitochondrial aspartyl-tRNA synthetase, and a highly characteristic pattern of white matter changes on MRI. A lactate peak on proton magnetic resonance spectroscopy (MRS) is often reported. LBSL presents as a clinical syndrome on a spectrum from mild to severe phenotypes [21, 22].

*DARS2* encodes mitochondrial aspartyl-tRNA synthetase (mtAspRS), the enzyme that attaches aspartate to the correct mitochondrial tRNA. This step is a necessary prerequisite in the translation of mitochondrial mRNA into functional protein. The *DARS2* mutation inheritance pattern is autosomal recessive, and strikingly, nearly all affected individuals have two compound heterozygous *DARS2* mutations, one of which is in most cases a splice site mutation in intron 2, upstream of exon 3 [6, 19]. The consequence of this mutation type is that exon 3 is not included in the mRNA, leading to a frameshift, premature stop, and absence of functional protein. However, these splice site mutations are suspected to be “leaky,” so that for some proportion of the mRNA formed, exon 3 is appropriately included and a normal full-length protein is produced [15]. The disease is very rare, but there is an especially high-carrier rate of *DARS2* mutations (1:95) in Finland, for reasons that have not yet been elucidated [23]. Over 60 different pathogenic *DARS2* mutations have been reported to date, and accordingly, there is great heterogeneity in the combinations of mutations that are causal in LBSL [22, 24, 25]. Although most LBSL cases are due to compound heterozygous *DARS2* mutations, a small subset of patients with homozygous *DARS2* mutations have been reported [7, 26–28].Thus, those carrying homozygous mutations do not necessarily die intrauterinely, but may present with a similar phenotype as patients carrying a compound heterozygous mutation.

Regarding the selective vulnerability of nervous system tracts in LBSL, analysis of intron 2 splice site mutations showed that the correct inclusion of exon 3 in the normal aspartyl-tRNA synthetase (mtAspRS) mRNA occurs much less efficiently in neural cells relative to other cell types [29]. In an elegant transgenic mouse model in which *DARS2* was depleted in either forebrain-hippocampal neurons or myelin-producing cells, it was shown that *DARS2* depletion in adult neurons leads to a significant mitochondrial dysfunction and progressive neuronal apoptosis [30]. On the other hand, oligodendrocytes deficient in *DARS2* were seemingly resistant to apoptosis despite dramatic respiratory chain deficiency, indicating that LBSL disease is predominantly due to neuronal pathology. Analysis of *DARS2* missense mutations on the expression, enzyme activity, localization, and dimerization of mtAspRS revealed varying effects on this enzyme’s properties. Most missense mutations resulted in at least a small reduction of mitochondrial aspartylation activity within human cells [15, 29]; however, the alteration of mtAspRs activity was not clearly correlated with disease severity. The implication for treatment is that it will be challenging to develop a single, common therapy to target the varied effects that the missense mutations impose on mtAspRS activity. Therefore, the more efficient approach would be to trial interventions that increase correct splicing of exon 3.

## Neuroimaging in LBSL

**Fig. 2:**
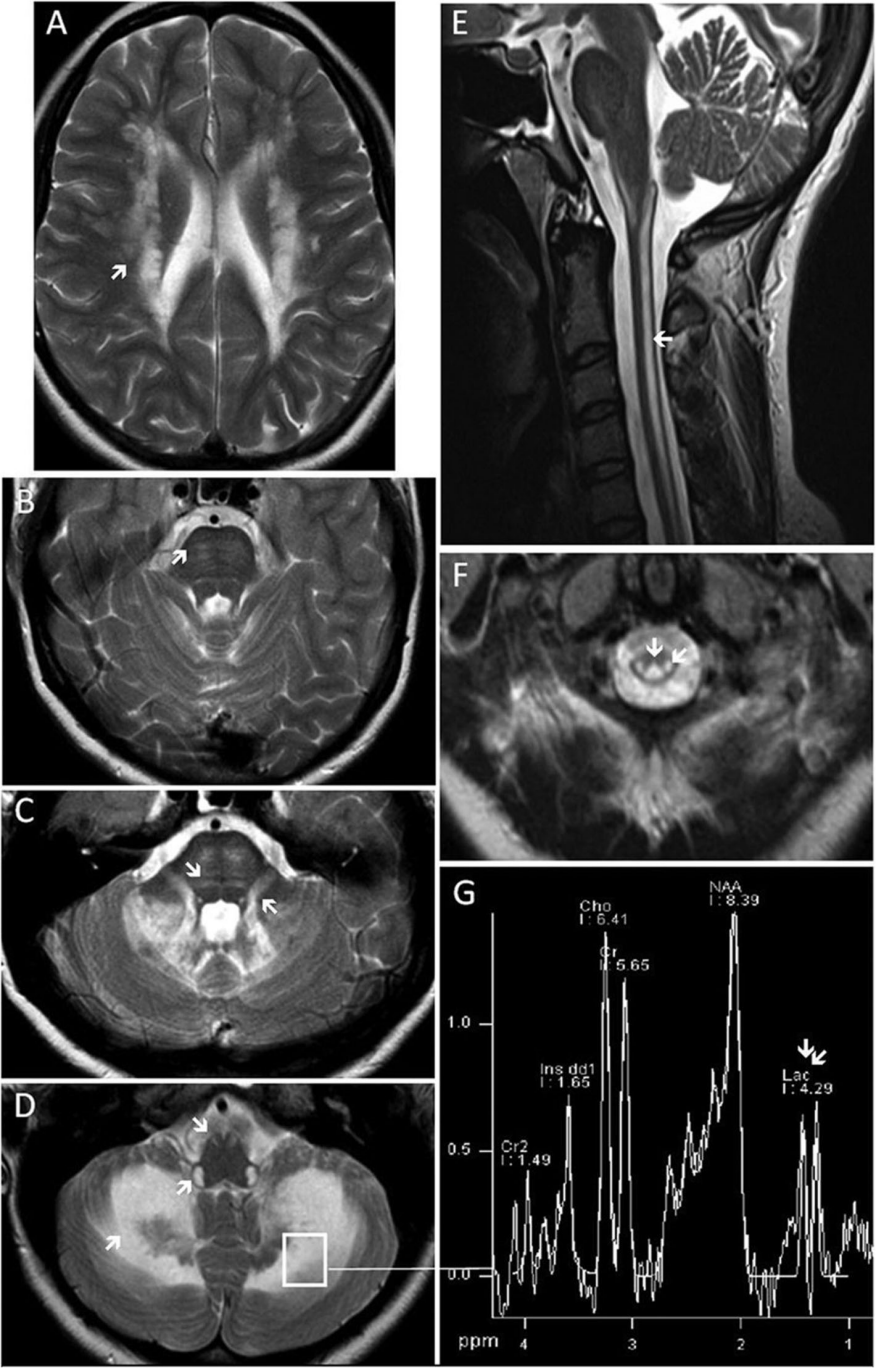
Brain and spinal cord T2-weighted MRI representative of LBSL. **a**–**d**, **f** Axial and **e** sagittal images show inhomogeneous signal abnormalities in the cerebral white matter (**a**), corticospinal tracts (**b**), superior cerebellar peduncles, medial lemniscus (**c**), pyramids, inferior cerebellar peduncles, cerebellar white matter (**d**), lateral corticospinal tracts, dorsal columns (**e**, **f**), and elevated lactate in magnetic resonance spectroscopy (**g**), thus meeting all major and some minor MRI criteria for LBSL (leukoencephalopathy with brainstem and spinal cord involvement and lactate elevation). Reprinted with permission [31].

There is a very characteristic pattern of supratentorial, infratentorial, and spinal cord white matter involvement in LBSL, in particular, there is high T2 signal “demarcation” of the involved neuroanatomical pathways. The cerebral and cerebellar white matter, corticospinal tracts, superior and/or inferior cerebellar peduncles, medial lemniscus, pyramids, lateral corticospinal tracts, and dorsal columns are most frequently affected (Fig. 2, Table 4). Most patients also have an MRS finding of lactate elevation in the abnormal white matter [6, 19, 20, 32]. It is not fully understood if LBSL results from myelin loss or a disruption of myelin development. Only one post-mortem examination of the brain has been done in a severely affected LBSL infant patient and showed widespread loss of myelin in the cerebral and cerebellar white matter with reactive astrocytes and foamy macrophages. Gray matter structures were preserved except for the globus pallidus [33]. A growing body of neuroimaging evidence suggests the lesions reflect a predominant demyelination [34, 35]. First, a demyelinating process may explain the lack of gray matter lesions. Diffusion-weighted imaging in a case of two affected sisters showed uniformly increased lesional water diffusion, suggesting increased extracellular water levels secondary to myelin degeneration [35]. In a case report of an LBSL patient who underwent serial imaging, compared to the initial MRI, restricted diffusion appeared at the lesion edges, where there was a dark ADC map, although at the center of the lesion the ADC signal had increased, which also suggests demyelination [26]. Furthermore, serial imaging of three LBSL patients demonstrated progressive loss of white matter volume only [31]. In magnetic resonance spectroscopy studies, the frontal and cerebellar white matter shows elevated lactate (Lac), reduced *N*-acetyl acetate (NAA), increased myoinositol (mI), and mildly elevated choline-containing compounds (Cho). Decrease in NAA and increase in mI in MRS suggests axonal damage or loss and gliosis, while elevation of Cho suggests low-grade demyelination [34].

There is evidence for an association of the MRI features and clinical phenotype, such that in late onset, mildly affected patients, the cerebral white matter abnormalities are less profound than in severely affected patients [22, 36–38]. However, it is unclear if the location or severity of MRI lesions is predictive of disease progression [39, 40].

## LBSL patterns of disease progression

The clinical severity in LBSL ranges from infantile onset, rapidly fatal disease to adult onset, slow and mild disease [22] (Table 2). However, the most common presentation of LBSL is slowly progressive deterioration of motor skills and in some cases cognitive skills beginning in childhood or adolescence [36]. In a longitudinal follow-up study of a large cohort of LBSL patients, the vast majority developed unsupported walking at an expected age. By adulthood, 50% required walking aids and 13% were wheelchair dependent. In general, earlier onset of symptoms predicted more severe neurological deterioration in the first decade after disease onset [22]. Case reports of affected siblings have shown that both mild and severe disease phenotypes can manifest even when the *DARS2* mutations are identifical [41, 42].

**Table 2:** Natural history of mt-aaRS-related leukodystrophies

	LBSL	LTBL	AARS2
Mean age at onset (age range)	8 years (5 months–40 years)	6 months (birth–16 months)	25 years (1.5–44 years)
Rate of disease progression	Slow, gradual > rapid decline	Biphasic with stability or improvement after early “hit”	Rapid decline within a few years
Risk of disability	Greater disability associated with earlier age of onset	Greater disability associated with initial clinical and imaging severity	High risk of motor and cognitive impairment
Risk of mortality	Increased for infantile-onset disease, otherwise low	Increased for early onset, severe disease with multi-organ involvement	Increased risk from late disease complications

The overall disease prevalence and frequency of key clinical features in LBSL is reported in Table 3. The lower limbs are primarily affected in LBSL. Leg spasticity, ataxia, hyperreflexia, weakness, and atrophy of the leg muscles have been reported for the majority of LBSL patients. Fine motor skills are generally less severely affected than ambulation [22, 36, 43]. Impaired proprioception, sphincter dysfunction, and urge incontinence are often present. Seizures occur in some patients, which is consistent with an increased frequency of epilepsy for many mitochondrial disorders [44, 45]. More recently, involvement of the visual system has been reported, including optic atrophy, hypoacusis, and diplopia [46]. Furthermore, exercise-induced ataxia and fever [7] and motor deterioration after minor head trauma or after infection have been described [26].

**Table 3:** Clinical features of mt-aaRS-related leukodystrophies

	LBSL (*n* = 128) % of cases reported	LTBL (*n* = 21) % of cases reported	AARS2 (*n* = 16) % of cases reported
Sex (male)	45	62	56
Cognitive impairment or learning problems	30	100	94
Motor or gait impairment	94	100	88
Seizures	7	43	8
Psychiatric/behavior problems	Not reported	n/a	93
Incontinence	4	n/a	0
Sensory changes	67	Not reported	13
Ovarian failure (female)	0	n/a	100
Cardiac disease	0	0	6
Visual/auditory impairment	5	14	6

Table 3 lists the number of cases of each disorder reported in the literature and the prevalence of the clinical signs of disease as a percentage of these cases.

Most patients have normal cognitive ability, although a higher proportion than expected among the general population require special education and serious intellectual disability has occasionally been reported [8, 19, 22]. A longitudinal study of a series of LBSL patients who had neuropsychological testing in adulthood revealed a cognitive profile for each that was similar to that reported in patients with multiple sclerosis [47], namely impairment of information-processing speed and working memory.

Few medications have been trialed to prevent LBSL disease progression or promote recovery from a metabolic crisis. Diplopia appeared to be steroid-responsive in a patient with adult-onset LBSL [46]. Exercise-induced paroxysmal gait ataxia and areflexia showed a dose-dependent sustained treatment response to a carbonic anhydrase inhibitor [7]. The drug cantharidin, a protein phosphatase 1 and 2A inhibitor and splicing modulator, has been shown to affect intron 2/exon 3 event splicing, which is the most common mutation in LBSL [22, 48]. Cantharidin itself is too toxic for human use, but less toxic variants and alternative protein phosphatase 1 or 2A inhibitors are under investigation [9, 49, 50].

## LTBL

**Fig. 3:**
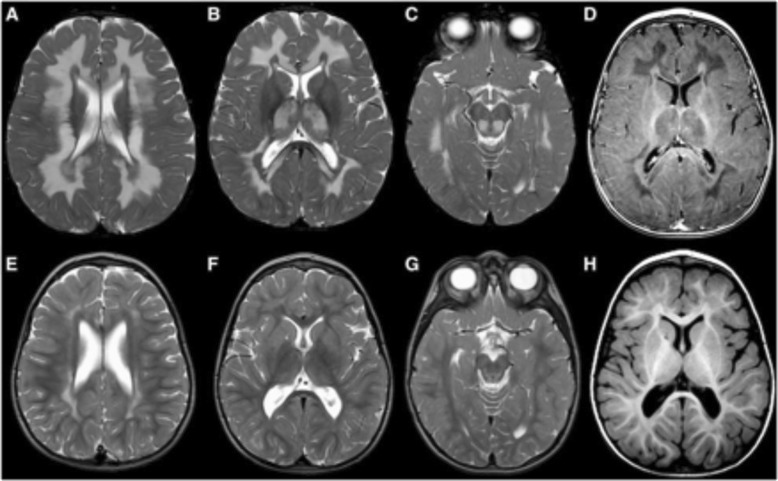
Brain T2-weighted MRI representative of mild LTBL. Axial T_2_- (**a**–**c** and **e**–**g**) and T_1_-weighted images (**d** and **h**) in (EARS2) patient 6 with a mild disease course at 11 months (**a**–**d**) and 3 years (**e**–**h**). Note the extensive T_2_-hyperintense and T_1_-hypointense signal of the deep cerebral white matter with sparing of a periventricular rim (**a**, **b**, and **d**). There are also signal abnormalities in the thalami (**b**) and dorsal part of the midbrain (**c**). Note the impressive improvement 2 years later (**e**–**h**). Reprinted with permission [45]

**Fig. 4:**
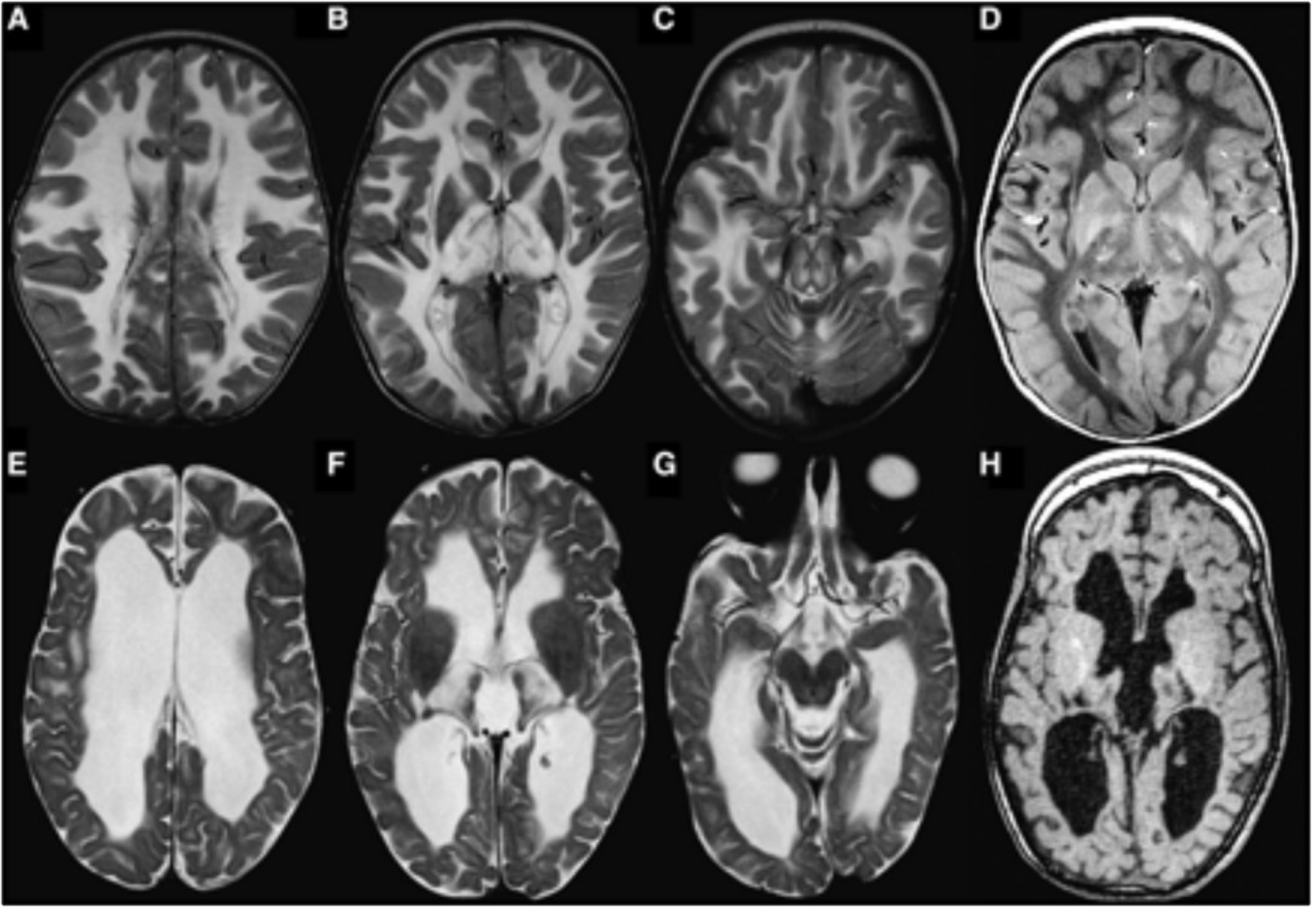
Brain T2-weighted MRI representative of severe LTBL. Axial T_2_- (**a**–**c** and **e**–**g**) and T_1_-weighted images (**d** and **h**) in (EARS2) patient 9 at 8 months (**a**–**d**) and 4 years (**e**–**h**). Note the diffuse T_2_-hyperintense and T_1_-hypointense signal of the cerebral white matter, only sparing a periventricular rim (**a**, **b**, and **d**). There are also signal abnormalities in the thalami (**b**) and the midbrain (**c**). Three years later, there is a serious atrophy of the cerebral white matter and thalami (**d**, **e**, and **h**). The midbrain signal abnormalities have disappeared (**f**). Reprinted with permission [45].

A detailed clinical and imaging review of leukoencephalopathy with thalamus and brainstem involvement and high lactate (LTBL) cases reveals both a mild and severe disease phenotype caused by either heterozygous or homozygous *EARS2* mutations, which encode mitochondrial glutamyl-tRNA synthetase [18, 51]. All patients typically experience infantile onset, rapidly progressive disease with severe MRI abnormalities and increased lactate in serum and proton magnetic resonance spectroscopy. Whereas mildly affected patients partially recover and make developmental progress over the next few years with associated MRI improvement and declining lactate levels, severely affected patients experience a stagnant clinical course, associated with MRI brain atrophy and persistently high lactate (Figs. 3 and 4).

In a study of biochemical assays of individual mitochondrial respiratory chain complexes (RCC) in cultured fibroblasts of LTBL patients, there was a spectrum of moderate reduction of RCC activity to virtually undetectable activity in some complexes (Fig. 1) [18, 31]. Thus, the authors proposed that the decreased cellular oxygen consumption rate and inversely increased lactate production is likely the effect of cumulative impairment of respiration by the whole set of mitochondrial respiratory chain complexes. Assays have not yet been performed in LTBL neuronal cells, which would permit insight into the effects of specific mutations on overall mt-aaRS activity in these especially vulnerable cells.

## LTBL neuroimaging

MRI pattern recognition paired with whole-exome sequencing first defined LTBL, which features extensive symmetrical cerebral white matter abnormalities sparing the periventricular rim and symmetrical signal abnormalities of the consistently affected corpus callosum, basal ganglia, thalami, midbrain, pons, medulla, and cerebellar white matter [10, 16, 18, 51] (Table 4). Some patients also exhibit dysplasia and/or agenesis or thinning of the posterior corpus callosum, and the cerebral white matter is initially more diffusely abnormal-appearing and edematous. Proton magnetic resonance spectroscopy shows increased lactate in affected brain regions. The lesional pathology in LTBL remains unclear, although imaging studies mostly support a process of delayed myelination. An early infantile case showed mildly elevated T2 and T1 signal in the subcortical white matter, indicating lack of myelin deposition. In subsequent studies, the T2 hyperintensity faded, indicating myelin deposition was occurring, albeit at a delayed timepoint [18]. Moreover, reports of cases with the absence of the thalami or parts of the corpus callosum support the theory that damage occurs to these structures antenatally, causing disruption of further development [52].

**Table 4:** Radiologic features of mt-aaRS-related leukodystrophies

	LBSL (*n* = 128) % of cases reported	LTBL (*n* = 21) % of cases reported	AARS2 (*n* = 16) % of cases reported
Imaging pattern			
Asymmetric	0	0	87
Symmetric	100	100	13
Distribution of T2/FLAIR signal abnormalities			
Cortical WM	100 (frontal, parietal or diffuse predominance)	100 (predominantly diffuse)	100 (fronto-parietal predominance)
Periventricular WM	100	9 (periventricular rim usually spared)	87
Subcortical WM	100	61	7 (rare subcortical U-fiber involvement)
Corpus callosum	79	95	100
Pyramidal tracts	99	14	81
Brainstem	100 (medullary tract predominance)	95	63
Cerebellum	97	83	27
Basal ganglia	8 (caudate or putamen)	62 (caudate, putamen, or globus pallidus)	39 (caudate)
Thalamus	0	100	Not reported
Lesion characteristics			
Diffusion restriction of any tract	82	38	87
Atrophy	3 (adult patients)	27	77
Elevated lactate on MR spectroscopy	83	71	20

Table 4 lists the prevalence of radiologic signs of disease based on percentage of cases reported in the literature.

## LTBL disease progression

In the mild LTBL variant, associated with either *EARS2* heterozgous or homozygous mutations, initially, there is a developmental delay, hypotonia, and poor feeding in infancy. Most patients experience early milestone regression prior to onset of clear, rapid neurological deterioration marked by spasticity and ataxia [10, 16, 18] (Tables 2 and 3). This phase is followed by partial recovery of lost motor and cognitive skills. The majority of patients have some residual spasticity perhaps requiring walking aids. Cognitive ability ranges from average to mild impairment [10, 16, 53]. As the clinical status improves, the MRI abnormalities also partially resolve.

A subset of LTBL patients experience onset of neurological impairment in the neonatal period, characterized by global failure to attain milestones, severe axial hypotonia, dysphagia, and often progression to spastic tetraparesis [18, 51, 52, 54]. There may be development of seizures. Although their symptoms stabilize over time, there is no clear recovery of neurologic function. Neuroimaging demonstrates more significant radiographic abnormalities, such as atrophy of the brain, brainstem or cerebellum, and corpus callosum dysgenesis, with associated high lactate on MRS. This severe phenotype has been associated with both homozygous and heterozygous EARS2 mutations. The progression of LTBL has also been characterized by early, rapid neurological deterioration, severe lactic acidosis, and multi-systems disease, which may lead to fatal cardiorespiratory failure [55, 56]. In the acute resuscitation phase, a standard treatment regimen for mitochondrial disorders has been administered, including thiamine, riboflavin, and coenzyme Q 10, but no long-term interventions have been developed.

## *AARS2*-related leukoencephalopathy

*AARS2* leukoencephalopathy is attributed to a defect in mitochondrial alanyl-tRNA synthetase, only compound heterozygous mutations have been reported [57–59]. The classic presentation of *AARS2* (ovario) leukoencephalopathy is a childhood-to-adulthood onset of neurologic deterioration with features of ataxia, spasticity, cognitive decline, and later in the disease course, frontal lobe impairment that manifests as psychiatric disorders or executive dysfunction. All affected female patients develop ovarian failure.

## *AARS2* neuroimaging

**Fig. 5:**
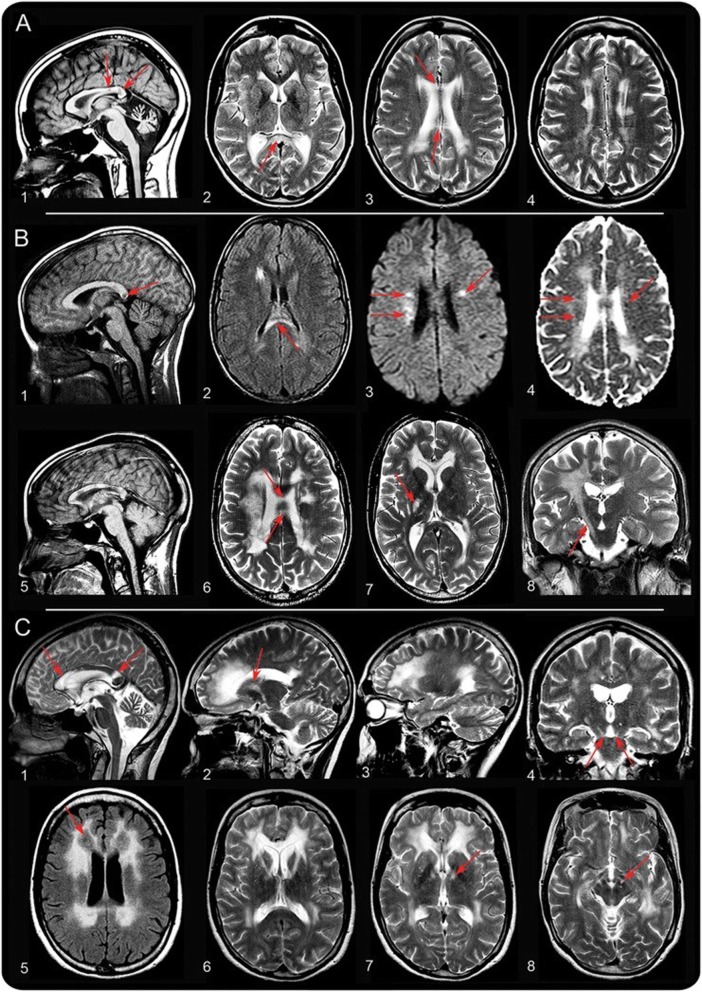
Brain and spinal cord T2-weighted MRI representative of AARS2-related leukodystrophy. **a** MRI in (AARS2 patient) P1 at age 28. The sagittal T1-weighted image shows serious cerebellar atrophy and 2 strips of abnormal signal in the splenium (arrows in image 1). The axial T2-weighted images show inhomogeneous areas of abnormal signal in the periventricular white matter. The areas on the left and right are connected signal abnormalities in the corpus callosum (arrows in images 2–4). **b** MRI in (AARS2 patient) P2 at age 14 (images 1 and 2), age 21 (images 3 and 4), and age 23 (images 5–8). At age 14, a lesion is seen in the splenium of the corpus callosum (arrow in image 1) and in the right frontal periventricular white matter. The diffusion-weighted images suggest the presence of multiple small areas of restricted diffusion in the abnormal white matter (arrows in image 3), confirmed by low signal of the corresponding areas on the apparent diffusion coefficient map (arrows in image 4). The most recent MRI shows multiple segments of abnormal signal in the corpus callosum (image 5 and arrows in image 6). More extensive signal abnormalities are seen in the periventricular white matter, especially on the right (images 6 and 7). Signal abnormalities extend downward through the posterior limb of the internal capsule and the pyramidal tracts in the brainstem on the right (arrows in images 7 and 8). **c** MRI in (AARS2 patient) P3 at age 35. The midsagittal image shows that the anterior part of the corpus callosum is abnormal, whereas only a strip of signal abnormality is seen in the splenium (arrows in image 1). Images 2 and 3 illustrate that the frontal and parietal white matter is abnormal, whereas the central white matter in between is normal. The tract involvement is evident (arrows in images 2 and 4). The axial fluid-attenuated inversion recovery image shows that the affected white matter is rarefied (arrow in image 5). The axial T2-weighted images illustrate the involvement of the anterior limb of the internal capsule (image 6) and the frontopontine tracts going down into the brainstem (arrows in images 7 and 8). Reprinted with permission [10]

The MRI typically shows a leukoencephalopathy with a significant involvement of left-right connections, descending tracts, and cerebellar atrophy [57, 60, 61]. MRI signal abnormalities are primarily found in frontal and parietal white matter, deep white matter, and the corpus callosum (Table 4, Fig. 5). Neuroimaging suggests that the pathology underlying white matter signal abnormalities is demyelination, although post-mortem tissue has not been studied. MR spectroscopy and diffusion-weighted imaging (DWI), reported only in one patient thus far, reflected the presence of active demyelination with an elevated lactate peak, high choline-containing compounds, and lesional DWI/ADC maps indicative of restricted diffusion [60]. Furthermore, the presence of spots of restricted diffusion in the cerebral white matter has a similar appearance to LBSL lesions and is ascribed to myelin vacuolization, which is commonly seen in mitochondrial leukodystrophies [57].

## *AARS2* leukoencephalopathy disease progression

The onset of symptoms usually occurs in the 3rd or 4th decade of life, starting with cognitive decline, mood, or behavioral problems then the development of signs consistent with frontal lobe dysfunction, such as stereotypies and apraxia, and motor impairment [57, 61] (Table 2). For affected women, ovarian failure is typically the first sign of disease. Spasticity is the most consistent finding and dystonia, dysarthria, or tremor may also be present. When the cerebellum is involved, ataxia or nystagmus appear. There may be rapidly progressive motor impairment with no evidence of cognitive regression or cognitive deterioration without motor dysfunction. Typically, within 5–10 years, these patients advance to the point of no or limited interaction with the environment, non-ambulatory status, and in many cases, premature death [58, 59, 62].

A smaller group of patients have exhibited motor or cognitive delays as early as infancy or childhood. In these cases, despite impaired balance, clumsiness, or learning difficulties, their clinical course remained stable until the teenage years, at which time rapid progression of motor, cognitive, and psychiatric impairment began [57]. One recent case presented with onset of bilateral optic atrophy and retinopathy in late infancy [63]. In addition to supratentorial and cerebellar multifocal white matter changes, there were signal changes in the dorsal columns of the spinal cord, a novel finding in this disease. The exam was notable for absent patellar reflexes and normal strength and sensation, but electromyogram revealed a motor demyelinating polyneuropathy.

Although it was previously thought that *AARS2* also causes an isolated, infantile onset cardiomyopathy without brain involvement, and one case has been reported with brain imaging signal abnormalities and atrophy as well as cardiomyopathy, lactic acidosis, and early death [64–67]. Therefore, investigation of the brain in infant patients who present with cardiomyopathy is worthwhile. The reported prevalence of key features of AARS2-leukodystrophy is listed in Table 3. No treatments for *AARS2* leukoencephalopathy have been systematically studied.

## Discussion

The disease patterns that are emerging for different mt-aaRS mutations, even in small cohorts of patients are quite distinct (Tables 2 and 3). The LBSL natural history generally features slowly progressive motor and cognitive deterioration after onset in childhood. The LTBL disease course is biphasic, involving one “hit” in the neonatal or infantile period with either stability or recovery based on the degree of MRI brain insult. *AARS2* leukoencephalopathy is characterized by relatively late onset of significant disease with rapid progression to motor and cognitive disability. Despite these differences, some common themes are present. Homozygous variants are present in each of these disorders and may manifest a disease phenotype anywhere along the spectrum. Siblings with the same set of mutations may be quite variably affected. Early onset of symptoms is often associated with more widespread or significant structural brain abnormalities. Accordingly, significant brain abnormalities are more often associated with faster neurological deterioration and ultimately greater disability. On the other hand, clinical recovery, if it occurs, is often paralleled by improvement in restricted diffusion, T2/FLAIR hyperintensity, and MRS lactate peak on serial imaging. Systemic involvement, especially in younger onset cases, is common and has important implications for morbidity and mortality.

It is not known what factors contribute to early and late onset forms of these disorders, but as previously noted, earlier disease onset is often correlated with more severe brain abnormalities that appear to have occurred early in development. Thus far, the rarity of the mt-aaRS diseases and high number of private mutations has precluded the establishment genotype-phenotype associations. Available evidence in a cohort of 66 LBSL patients suggests that the genotype influences the phenotype; in particular, some combinations of mutations were consistently associated with a mild phenotype, but larger numbers of patients are needed to confirm this [22]. One theory is that some combinations of the two compound heterozygous mutations may lead to a more profound loss of mt-aaRS enzyme activity than others, leading to greater mitochondrial respiratory chain complex dysfunction. Given that carriers are asymptomatic and many affected patients have symptoms that do not affect their survival or mobility until after young adulthood, it is plausible that compound heterozygous mutations are the primary inheritance mode.

As illustrated in Fig. 1, decreased EARS2 and AARS2 enzyme activity in cultured cells leads to decreased function of specific mitochondrial respiratory chain complexes, presumably because of misfolding of RCC proteins. Furthermore, in LTBL, there are measured decreases in the cellular oxygen consumption rate. Therefore, the leading theory is that the increased lactate production is *secondary* to these cumulative defects in mitochondrial respiration. Indeed, the finding of lactate elevation is not required for diagnosis of any of these disorders, and although the majority of patients exhibit elevated lactate on MRS, the levels appear to fluctuate over time. Furthermore, lactate elevation is known to be a non-specific marker of mitochondrial disease [68].

Beyond their canonical function of directly altering tRNA charging, thereby facilitating the translation of proteins that form the mitochondrial respiratory chain complexes (Fig. 1), it is also possible that the mt-aaRS affect non-canonical biochemical pathways in neurons, such as those involved in cell signalling, transcription, or rRNA biogenesis [69, 70]. In humans, two sets of distinct nuclear genes code for either the cytosolic aaRSs or the mitochondrial aaRSs [71]. Cytosolic aaRSs have roles in numerous non-canonical cellular functions beyond translation, such as angiogenesis, immune responses, inflammation, tumorigenesis, and neuronal development [72, 73]. Similarly, reports of non-canonical roles for the mt-aaRS are beginning to emerge, such as a pro-angiogenic function for rat mitochondrial tryptophanyl-tRNA synthetase (WARS2) [74] and a possible role for mt-aaRS mutations in the integrated stress response (ISR) [75]. The ISR is a highly conserved homeostatic program in eukaryotic cells that is activated in response to a diversity of pathologic states or cellular stresses. Agnew et al. postulated that activation of the ISR through alternative mechanisms is dependent on the degree of mitochondrial translation inhibition [75]. Complete inhibition of mitochondrial translation in *DARS2* knockout mice results in the accumulation of unassembled nuclear-encoded respiratory chain subunits, causing severe proteostatic stress and UPR^mt^-dependent ISR activation [76]. In this model, there is failure of the ISR to achieve homeostasis, resulting in severe cardiac disease and decreased survival. However, *partial* inhibition of mitochondrial translation in a *WARS2* mouse model leads to ISR activation due to respiratory chain dysfunction and loss of mitochondrial membrane potential, resulting in a more mild cardiac phenotype and increased survival [75].

Further evidence that malfunction of the mitochondrial translational machinery promotes pathologic ISR activation in white matter disease comes from the study of disease mechanisms in Vanishing White Matter Disease (VWMD). VWMD is a severe progressive leukodystrophy with episodic clinical deterioration due to mutations in the five genes that encode the subunits of the elongation initiation factor complex EIF2B [77–80]. EIF2B is a key regulator of mRNA translation [81, 82]. Interestingly, EIF2B is also a regulator of the ISR and abnormal ISR response is thought to be the main underlying pathomechanism in VWMD [83–85]. Many EIF2B mutations in VWMD have no clear effect on the enzyme’s activity or complex formation [86–88]. However, some VWMD mutations destabilize the decameric eIF2B holoenzyme and impair its enzymatic activity. In a mouse VWMD model, an ISR inhibitor (ISRIB) blocked ISR activation, stabilized VWMD mutant eIF2B in the decameric form, and restored normal catalytic activity [89]. Similar to the mt-aaRS disorders, it is unclear why VWMD selectively affects the white matter [90]. Recent investigations have shown that VWMD astrocytes appear toxic to neurons and oligodendrocytes and show impaired maturation [91, 92]. Mutation of EIF2B in VWMD mutant mice resulted in ISR upregulation in both astrocytes and oligodendrocytes that preceded myelin loss and motor deficits [93]. Such mechanistic studies have not yet been attempted in the mt-aaRS leukodystrophies.

## Conclusion

The significant clinical variability and tissue specificity found within each mt-aaRS disorder highlight the importance of understanding the factors influencing mitochondrial translation in different cell types. Overall, the effects of mt-aaRS mutations on the process of translation may be subtle and difficult to dissociate, especially given the high number of private mutations. Furthermore, there is a growing rationale to explore potential non-canonical roles of the mt-aaRSs in immune regulation, inflammation, and neuronal differentiation. Mechanistic studies are challenging, especially since it appears that some of the mutation effects are specific to a neuronal or glial context, and obtaining patient cells that can be cultured for this purpose is cost and labor intensive.

## Data Availability

Data sharing is not applicable to this article as no datasets were generated or analyzed during the current study.

## Abbreviations

Cho: Choline-containing compounds
ISR: Integrated stress response
ISRIB: ISR inhibitor
LBSL: Leukoencephalopathy with brainstem and spinal cord involvement and high lactate
LTBL: Leukoencephalopathy with thalamus and brainstem involvement and high lactate
Mi: Myoinositol
MRR: Mitochondrial respiratory rate
MRS: Magnetic resonance spectroscopy
mt-aaRSs: Mitochondrial aminoacyl-tRNA synthetase proteins
mtAspRS: Mitochondrial aspartyl-tRNA synthetase
NAA: *N*-acetyl acetate
OCR: Oxygen consumption rate
RCC: Respiratory chain complex
VWMD: Vanishing White Matter Disease
WARS2: Mitochondrial tryptophanyl-tRNA synthetase

## References

[1] Boczonadi V, Ricci G, Horvath R (2018). Mitochondrial DNA transcription and translation: clinical syndromes. Essays Biochem..

[2] Gorman GS, Chinnery PF, DiMauro S, Hirano M, Koga Y, McFarland R (2016). Mitochondrial diseases. Nat Rev Dis Primers..

[3] Ognjenovic J, Simonovic M (2018). Human aminoacyl-tRNA synthetases in diseases of the nervous system. RNA biology..

[4] Meyer-Schuman R, Antonellis A (2017). Emerging mechanisms of aminoacyl-tRNA synthetase mutations in recessive and dominant human disease. Hum Mol Genet..

[5] Sissler M, Gonzalez-Serrano LE, Westhof E (2017). Recent advances in mitochondrial aminoacyl-tRNA synthetases and disease. Trends in molecular medicine..

[6] Scheper GC, van der Klok T, van Andel RJ, van Berkel CG, Sissler M, Smet J (2007). Mitochondrial aspartyl-tRNA synthetase deficiency causes leukoencephalopathy with brain stem and spinal cord involvement and lactate elevation. Nature genetics..

[7] Synofzik M, Schicks J, Lindig T, Biskup S, Schmidt T, Hansel J (2011). Acetazolamide-responsive exercise-induced episodic ataxia associated with a novel homozygous DARS2 mutation. Journal of medical genetics..

[8] Mikhailova SV, Zakharova E, Banin AV, Demushkina AA, Petrukhin AS (2009). Clinical and molecular genetic diagnosis of leukoencephalopathy with brainstem and spinal cord involvement and lactate elevation in children. Zhurnal nevrologii i psikhiatrii imeni SS Korsakova..

[9] Xu JG, Zhang JH, Chen JW (2011). Study on the change of the content of cantharidin in Mylabris befere and after biortransfermation. Zhong Yao Cai.

[10] Taskin BD, Karalok ZS, Gurkas E, Aydin K, Aydogmus U, Ceylaner S (2016). Early-onset mild type leukoencephalopathy caused by a homozygous EARS2 mutation. Journal of child neurology..

[11] Moulinier L, Ripp R, Castillo G, Poch O, Sissler M (2017). MiSynPat: an integrated knowledge base linking clinical, genetic, and structural data for disease-causing mutations in human mitochondrial aminoacyl-tRNA synthetases. Hum Mutat..

[12] Brandon MC, Lott MT, Nguyen KC, Spolim S, Navathe SB, Baldi P (2005). MITOMAP: a human mitochondrial genome database--2004 update. Nucleic acids research..

[13] Webb BD, Wheeler PG, Hagen JJ, Cohen N, Linderman MD, Diaz GA (2015). Novel, compound heterozygous, single-nucleotide variants in MARS2 associated with developmental delay, poor growth, and sensorineural hearing loss. Hum Mutat..

[14] Bayat V, Thiffault I, Jaiswal M, Tetreault M, Donti T, Sasarman F (2012). Mutations in the mitochondrial methionyl-tRNA synthetase cause a neurodegenerative phenotype in flies and a recessive ataxia (ARSAL) in humans. PLoS biology..

[15] van Berge L, Dooves S, van Berkel CG, Polder E, van der Knaap MS, Scheper GC (2012). Leukoencephalopathy with brain stem and spinal cord involvement and lactate elevation is associated with cell-type-dependent splicing of mtAspRS mRNA. The Biochemical journal..

[16] Biancheri R, Lamantea E, Severino M, Diodato D, Pedemonte M, Cassandrini D (2015). Expanding the clinical and magnetic resonance spectrum of leukoencephalopathy with thalamus and brainstem involvement and high lactate (LTBL) in a Patient Harboring a Novel EARS2 Mutation. JIMD reports..

[17] Hausmann CD, Ibba M (2008). Aminoacyl-tRNA synthetase complexes: molecular multitasking revealed. FEMS microbiology reviews..

[18] Steenweg ME, Ghezzi D, Haack T, Abbink TE, Martinelli D, van Berkel CG (2012). Leukoencephalopathy with thalamus and brainstem involvement and high lactate 'LTBL' caused by EARS2 mutations. Brain.

[19] Lin J, Chiconelli Faria E, Da Rocha AJ, Rodrigues Masruha M, Pereira Vilanova LC, Scheper GC (2010). Leukoencephalopathy with brainstem and spinal cord involvement and normal lactate: a new mutation in the DARS2 gene. J Child Neurol..

[20] van der Knaap MS, Salomons GS, Adam MP, Ardinger HH, Pagon RA, Wallace SE, LJH B, Stephens K (1993). Leukoencephalopathy with brain stem and spinal cord involvement and lactate elevation.

[21] Finsterer J, Zarrouk-Mahjoub S (2017). Phenotypic spectrum of DARS2 mutations. Journal of the neurological sciences..

[22] van Berge L, Hamilton EM, Linnankivi T, Uziel G, Steenweg ME, Isohanni P (2014). Leukoencephalopathy with brainstem and spinal cord involvement and lactate elevation: clinical and genetic characterization and target for therapy. Brain.

[23] Isohanni P, Linnankivi T, Buzkova J, Lonnqvist T, Pihko H, Valanne L (2010). DARS2 mutations in mitochondrial leucoencephalopathy and multiple sclerosis. Journal of medical genetics..

[24] Shimojima K, Higashiguchi T, Kishimoto K, Miyatake S, Miyake N, Takanashi JI (2017). A novel DARS2 mutation in a Japanese patient with leukoencephalopathy with brainstem and spinal cord involvement but no lactate elevation. Hum Genome Variation..

[25] Yelam A, Nagarajan E, Chuquilin M, Govindarajan R. Leucoencephalopathy with brain stem and spinal cord involvement and lactate elevation: a novel mutation in the DARS2 gene. BMJ case reports. 2019;12(1). Epub 2019/01/13. 10.1136/bcr-2018-227755 PMC6340551 30635318

[26] Kohler C, Heyer C, Hoffjan S, Stemmler S, Lucke T, Thiels C (2015). Early-onset leukoencephalopathy due to a homozygous missense mutation in the DARS2 gene. Molecular and cellular probes..

[27] Yamashita S, Miyake N, Matsumoto N, Osaka H, Iai M, Aida N (2013). Neuropathology of leukoencephalopathy with brainstem and spinal cord involvement and high lactate caused by a homozygous mutation of DARS2. Brain Dev..

[28] Miyake N, Yamashita S, Kurosawa K, Miyatake S, Tsurusaki Y, Doi H (2011). A novel homozygous mutation of DARS2 may cause a severe LBSL variant. Clinical genetics..

[29] van Berge L, Kevenaar J, Polder E, Gaudry A, Florentz C, Sissler M (2013). Pathogenic mutations causing LBSL affect mitochondrial aspartyl-tRNA synthetase in diverse ways. Biochem J..

[30] Aradjanski M, Dogan SA, Lotter S, Wang S, Hermans S, Wibom R (2017). DARS2 protects against neuroinflammation and apoptotic neuronal loss, but is dispensable for myelin producing cells. Human molecular genetics..

[31] Steenweg ME, Vanderver A, Ceulemans B, Prabhakar P, Regal L, Fattal-Valevski A (2012). Novel infantile-onset leukoencephalopathy with high lactate level and slow improvement. Archives of neurology..

[32] Kassem H, Wafaie A, Abdelfattah S, Farid T (2014). Leukoencephalopathy with brainstem and spinal cord involvement and lactate elevation (LBSL): assessment of the involved white matter tracts by MRI. Eur J Radiol..

[33] Steenweg ME, van Berge L, van Berkel CG, de Coo IF, Temple IK, Brockmann K (2012). Early-onset LBSL: how severe does it get?. Neuropediatrics..

[34] Steenweg ME, Pouwels PJ, Wolf NI, van Wieringen WN, Barkhof F, van der Knaap MS (2011). Leucoencephalopathy with brainstem and spinal cord involvement and high lactate: quantitative magnetic resonance imaging. Brain : a journal of neurology..

[35] Tzoulis C, Tran GT, Gjerde IO, Aasly J, Neckelmann G, Rydland J (2012). Leukoencephalopathy with brainstem and spinal cord involvement caused by a novel mutation in the DARS2 gene. J Neurol..

[36] van der Knaap MS, van der Voorn P, Barkhof F, Van Coster R, Krageloh-Mann I, Feigenbaum A (2003). A new leukoencephalopathy with brainstem and spinal cord involvement and high lactate. Annals of neurology..

[37] Linnankivi T, Lundbom N, Autti T, Hakkinen AM, Koillinen H, Kuusi T (2004). Five new cases of a recently described leukoencephalopathy with high brain lactate. Neurology..

[38] Tavora DG, Nakayama M, Gama RL, Alvim TC, Portugal D, Comerlato EA (2007). Leukoencephalopathy with brainstem and spinal cord involvement and high brain lactate: report of three Brazilian patients. Arquivos de neuro-psiquiatria..

[39] Tillema JM, Derks MG, Pouwels PJ, de Graaf P, van Rappard DF, Barkhof F (2015). Volumetric MRI data correlate to disease severity in metachromatic leukodystrophy. Annals of clinical and translational neurology..

[40] Loes DJ, Fatemi A, Melhem ER, Gupte N, Bezman L, Moser HW (2003). Analysis of MRI patterns aids prediction of progression in X-linked adrenoleukodystrophy. Neurology..

[41] Yahia A, Elsayed L, Babai A, Salih MA, El-Sadig SM, Amin M (2018). Intra-familial phenotypic heterogeneity in a Sudanese family with DARS2-related leukoencephalopathy, brainstem and spinal cord involvement and lactate elevation: a case report. BMC neurology..

[42] Labauge P, Dorboz I, Eymard-Pierre E, Dereeper O, Boespflug-Tanguy O (2011). Clinically asymptomatic adult patient with extensive LBSL MRI pattern and DARS2 mutations. Journal of neurology..

[43] Schicks J, Schols L, van der Knaap MS, Synofzik M (2013). Teaching NeuroImages: MRI guides genetics: leukoencephalopathy with brainstem and spinal cord involvement (LBSL). Neurology..

[44] Finsterer J, Zarrouk MS (2012). Epilepsy in mitochondrial disorders. Seizure..

[45] Finsterer J, Mahjoub SZ (2013). Presentation of adult mitochondrial epilepsy. Seizure..

[46] Cheng FB, Shen PP, Zhou HW, Meng HM, Yang Y, Feng JC (2013). Adult-onset leukoencephalopathy with brain stem and spinal cord involvement in Chinese Han population: a case report and literature review. Neurology India..

[47] Martikainen MH, Ellfolk U, Majamaa K (2013). Impaired information-processing speed and working memory in leukoencephalopathy with brainstem and spinal cord involvement and elevated lactate (LBSL) and DARS2 mutations: a report of three adult patients. Journal of neurology..

[48] Novoyatleva T, Heinrich B, Tang Y, Benderska N, Butchbach ME, Lorson CL (2008). Protein phosphatase 1 binds to the RNA recognition motif of several splicing factors and regulates alternative pre-mRNA processing. Human molecular genetics..

[49] Moed L, Shwayder TA, Chang MW (2001). Cantharidin revisited: a blistering defense of an ancient medicine. Archives of dermatology..

[50] Han L, Sun YJ, Pan YF, Ding H, Chen X, Zhang X (2014). Cantharidin combined with chemotherapy for Chinese patients with metastatic colorectal cancer. Asian Pacific journal of cancer prevention : APJCP..

[51] Talim B, Pyle A, Griffin H, Topaloglu H, Tokatli A, Keogh MJ (2013). Multisystem fatal infantile disease caused by a novel homozygous EARS2 mutation. Brain.

[52] Kevelam SH, Klouwer FC, Fock JM, Salomons GS, Bugiani M, van der Knaap MS (2016). Absent thalami caused by a homozygous EARS2 mutation: expanding disease spectrum of LTBL. Neuropediatrics..

[53] Gungor O, Ozkaya AK, Sahin Y, Gungor G, Dilber C, Aydin K (2016). A compound heterozygous EARS2 mutation associated with mild leukoencephalopathy with thalamus and brainstem involvement and high lactate (LTBL). Brain & development..

[54] Oliveira R, Sommerville EW, Thompson K, Nunes J, Pyle A, Grazina M (2017). Lethal neonatal LTBL associated with biallelic EARS2 variants: case report and review of the reported neuroradiological features. JIMD reports..

[55] Danhauser K, Haack TB, Alhaddad B, Melcher M, Seibt A, Strom TM (2016). EARS2 mutations cause fatal neonatal lactic acidosis, recurrent hypoglycemia and agenesis of corpus callosum. Metabolic brain disease..

[56] Sellars EA, Balmakund T, Bosanko K, Nichols BL, Kahler SG, Zarate YA (2017). Severe metabolic acidosis and hepatopathy due to leukoencephalopathy with thalamus and brainstem involvement and high lactate. Neuropediatrics..

[57] Dallabona C, Diodato D, Kevelam SH, Haack TB, Wong LJ, Salomons GS (2014). Novel (ovario) leukodystrophy related to AARS2 mutations. Neurology..

[58] Hamatani M, Jingami N, Tsurusaki Y, Shimada S, Shimojima K, Asada-Utsugi M (2016). The first Japanese case of leukodystrophy with ovarian failure arising from novel compound heterozygous AARS2 mutations. Journal of human genetics..

[59] Szpisjak L, Zsindely N, Engelhardt JI, Vecsei L, Kovacs GG, Klivenyi P (2017). Novel AARS2 gene mutation producing leukodystrophy: a case report. J Hum Genet..

[60] Taglia I, Di Donato I, Bianchi S, Cerase A, Monti L, Marconi R (2018). AARS2-related ovarioleukodystrophy: clinical and neuroimaging features of three new cases. Acta neurologica Scandinavica..

[61] Lakshmanan R, Adams ME, Lynch DS, Kinsella JA, Phadke R, Schott JM (2017). Redefining the phenotype of ALSP and AARS2 mutation-related leukodystrophy. Neurol Genet..

[62] Dong Q, Long L, Chang YY, Lin YJ, Liu M, Lu ZQ (2018). An adolescence-onset male leukoencephalopathy with remarkable cerebellar atrophy and novel compound heterozygous AARS2 gene mutations: a case report. J Hum Genet..

[63] Peragallo JH, Keller S, van der Knaap MS, Soares BP, Shankar SP (2018). Retinopathy and optic atrophy: expanding the phenotypic spectrum of pathogenic variants in the AARS2 gene. Ophthalmic genetics..

[64] Kamps R, Szklarczyk R, Theunissen TE, Hellebrekers D, Sallevelt S, Boesten IB (2018). Genetic defects in mtDNA-encoded protein translation cause pediatric, mitochondrial cardiomyopathy with early-onset brain disease. Eur J Hum Genet.

[65] Sommerville EW, Zhou XL, Olahova M, Jenkins J, Euro L, Konovalova S (2019). Instability of the mitochondrial alanyl-tRNA synthetase underlies fatal infantile-onset cardiomyopathy. Human molecular genetics..

[66] Mazurova S, Magner M, Kucerova-Vidrova V, Vondrackova A, Stranecky V, Pristoupilova A (2017). Thymidine kinase 2 and alanyl-tRNA synthetase 2 deficiencies cause lethal mitochondrial cardiomyopathy: case reports and review of the literature. Cardiology in the young..

[67] Gotz A, Tyynismaa H, Euro L, Ellonen P, Hyotylainen T, Ojala T (2011). Exome sequencing identifies mitochondrial alanyl-tRNA synthetase mutations in infantile mitochondrial cardiomyopathy. American journal of human genetics..

[68] Haas RH, Parikh S, Falk MJ, Saneto RP, Wolf NI, Darin N (2008). The in-depth evaluation of suspected mitochondrial disease. Mol Genet Metab..

[69] Antonellis A, Green ED (2008). The role of aminoacyl-tRNA synthetases in genetic diseases. Annu Rev Genomics Hum Genet..

[70] Brown MV, Reader JS, Tzima E (2010). Mammalian aminoacyl-tRNA synthetases: cell signaling functions of the protein translation machinery. Vasc Pharmacol..

[71] Bonnefond L, Fender A, Rudinger-Thirion J, Giege R, Florentz C, Sissler M (2005). Toward the full set of human mitochondrial aminoacyl-tRNA synthetases: characterization of AspRS and TyrRS. Biochemistry..

[72] Guo M, Schimmel P, Yang XL (2010). Functional expansion of human tRNA synthetases achieved by structural inventions. FEBS letters..

[73] Kim S, You S, Hwang D (2011). Aminoacyl-tRNA synthetases and tumorigenesis: more than housekeeping. Nature reviews Cancer..

[74] Wang M, Sips P, Khin E, Rotival M, Sun X, Ahmed R (2016). Wars2 is a determinant of angiogenesis. Nat Commun..

[75] Agnew T, Goldsworthy M, Aguilar C, Morgan A, Simon M, Hilton H (2018). A Wars2 mutant mouse model displays OXPHOS deficiencies and activation of tissue-specific stress response pathways. Cell Rep..

[76] Dogan SA, Pujol C, Maiti P, Kukat A, Wang S, Hermans S (2014). Tissue-specific loss of DARS2 activates stress responses independently of respiratory chain deficiency in the heart. Cell metabolism..

[77] Schiffmann R, Fogli A, van der Knaap MS, Boespflug-Tanguy O, Adam MP, Ardinger HH, Pagon RA, Wallace SE, LJH B, Stephens K (1993). Childhood ataxia with central nervous system hypomyelination/Vanishing White Matter.

[78] van der Knaap MS, Barth PG, Gabreels FJ, Franzoni E, Begeer JH, Stroink H (1997). A new leukoencephalopathy with vanishing white matter. Neurology..

[79] Leegwater PA, Vermeulen G, Konst AA, Naidu S, Mulders J, Visser A (2001). Subunits of the translation initiation factor eIF2B are mutant in leukoencephalopathy with vanishing white matter. Nature genetics..

[80] Wortham NC, Proud CG (2015). Biochemical effects of mutations in the gene encoding the alpha subunit of eukaryotic initiation factor (eIF) 2B associated with Vanishing White Matter disease. BMC Med Genet..

[81] Yang W, Hinnebusch AG (1996). Identification of a regulatory subcomplex in the guanine nucleotide exchange factor eIF2B that mediates inhibition by phosphorylated eIF2. Molecular and cellular biology..

[82] Hinnebusch AG, Lorsch JR. The mechanism of eukaryotic translation initiation: new insights and challenges. Cold Spring Harbor perspectives in biology. 2012;4(10). Epub 2012/07/21. 10.1101/cshperspect.a011544 PMC3475172 22815232

[83] Sekine Y, Zyryanova A, Crespillo-Casado A, Fischer PM, Harding HP, Ron D (2015). Stress responses. Mutations in a translation initiation factor identify the target of a memory-enhancing compound. Science..

[84] Sidrauski C, Tsai JC, Kampmann M, Hearn BR, Vedantham P, Jaishankar P (2015). Pharmacological dimerization and activation of the exchange factor eIF2B antagonizes the integrated stress response. eLife..

[85] van Kollenburg B, Thomas AA, Vermeulen G, Bertrand GA, van Berkel CG, Pronk JC (2006). Regulation of protein synthesis in lymphoblasts from vanishing white matter patients. Neurobiol Dis..

[86] Liu R, van der Lei HD, Wang X, Wortham NC, Tang H, van Berkel CG (2011). Severity of vanishing white matter disease does not correlate with deficits in eIF2B activity or the integrity of eIF2B complexes. Hum Mutat..

[87] Fogli A, Schiffmann R, Hugendubler L, Combes P, Bertini E, Rodriguez D (2004). Decreased guanine nucleotide exchange factor activity in eIF2B-mutated patients. European journal of human genetics : EJHG..

[88] Li W, Wang X, Van Der Knaap MS, Proud CG (2004). Mutations linked to leukoencephalopathy with vanishing white matter impair the function of the eukaryotic initiation factor 2B complex in diverse ways. Mol Cell Biol..

[89] Wong YL, LeBon L, Edalji R, Lim HB, Sun C, Sidrauski C. The small molecule ISRIB rescues the stability and activity of Vanishing White Matter Disease eIF2B mutant complexes. eLife. 2018;7 Epub 2018/03/01. 10.7554/eLife.32733 PMC5829914 29489452

[90] Bugiani M, Boor I, Powers JM, Scheper GC, van der Knaap MS (2010). Leukoencephalopathy with vanishing white matter: a review. Journal of neuropathology and experimental neurology..

[91] Dooves S, Bugiani M, Postma NL, Polder E, Land N, Horan ST (2016). Astrocytes are central in the pathomechanisms of vanishing white matter. J Clin Investig..

[92] Bugiani M, Boor I, van Kollenburg B, Postma N, Polder E, van Berkel C (2011). Defective glial maturation in vanishing white matter disease. Journal of neuropathology and experimental neurology..

[93] Wong YL, LeBon L, Basso AM, Kohlhaas KL, Nikkel AL, Robb HM, et al. eIF2B activator prevents neurological defects caused by a chronic integrated stress response. eLife. 2019;8 Epub 2019/01/10. 10.7554/eLife.42940 PMC6326728 30624206

